# Clinical Utility of Optical Genome Mapping as an Additional Tool in a Standard Cytogenetic Workup in Hematological Malignancies

**DOI:** 10.3390/cancers17091436

**Published:** 2025-04-25

**Authors:** Gokce A. Toruner, Shimin Hu, Sanam Loghavi, Chi Young OK, Zhenya Tang, Qing Wei, Rashmi Kanagal-Shamanna, L. Jeffrey Medeiros, Guilin Tang

**Affiliations:** 1 Department of Hematopathology, The University of Texas MD Anderson Cancer Center, Houston, TX 77030, USA; 2 Department of Pathology & Microbiology, University of Nebrasks Medical Center, Omaha, NE 68198, USA

**Keywords:** optical genome mapping, standard cytogenetics workup, hematological malignancies, clinical utility, analytical sensitivity

## Abstract

Background and Objective: The primary objective of this study is to evaluate the added value of optical genome mapping (OGM) when integrated into the standard cytogenetic workup (SCGW) for hematological malignancies. Methods: The study cohort comprised 519 cases with different types of hematological malignancies. OGM and SCGW (including G-banded karyotyping and fluorescence in situ hybridization) were performed on blood and/or bone marrow. The analytical sensitivity of OGM, defined as the detection of all additional cytogenomic aberrations, and its clinical utility, referring to aberrations with diagnostic, prognostic, or therapeutic significance, were assessed. Results: OGM led to increased analytical sensitivity and clinical utility in 58% and 15% of the cases, respectively. The clinical utility varied across different malignancies, with the highest utility in T-lymphoblast leukemia (52%), followed by mixed phenotype acute leukemia (43%), B-lymphoblastic leukemia (37%), other B-cell lymphomas (22%), mature T-cell leukemia/lymphoma (20%), chronic lymphocytic leukemia (14%), acute myeloid leukemia (13%), multiple myeloma (13%), mantle cell lymphoma (8%), myelodysplastic/myeloproliferative neoplasms (6%), myelodysplastic syndrome (5%), and myeloproliferative neoplasms (0%). Conclusion: Compared to SCGW, OGM detects additional cytogenomic aberrations in approximately 58% of cases. OGM provides clinical utility at varying rates across different types of hematological malignancies. Given these differences, strategic triaging can help maximize the clinical value of OGM by focusing on diseases where it offers the most significant benefit.

## Simple Summary

Optical genome mapping (OGM) is increasingly utilized in clinical cytogenetics laboratories; however, systematic studies assessing its added value compared to the standard cytogenetics work-up (SCGW) remain limited. In this single-institution study of 519 hematological malignancies, we evaluated the additional contributions of OGM beyond SCGW. OGM identified additional cytogenomic abnormalities in 58% of cases, with 15% of cases showing findings that impacted diagnosis, prognosis, or treatment decisions. However, the clinical utility of OGM varied across hematological malignancies. For instance, OGM detected clinically significant additional findings in more than half of T-lymphoblastic leukemia cases, whereas none of the myeloproliferative neoplasms demonstrated additional actionable results. Given these differences, we recommend that clinical laboratories triage and prioritize OGM for diseases where it provides the most significant benefit, particularly when resources are limited.

## 1. Introduction

G-banded chromosomal analysis (karyotyping) and fluorescence in situ hybridization (FISH) have traditionally been the main tools in clinical cytogenetics laboratories for standard cytogenetic work-up (SCGW). Karyotyping, while cost-effective and broadly informative, has limitations like low resolution (6–10 Mb) and a reliance on tissue culture. Despite these constraints, as a single-cell assay, its ability to provide a detailed look at clonal architecture is invaluable in cancer genetics. FISH was developed to complement the limitations of karyotyping. It offers better resolution and specificity, detecting chromosomal aberrations that karyotyping might miss. However, since FISH is designed to detect specific genetic abnormalities, it is not broad enough to capture the whole genome. Using both FISH and karyotyping provides a balanced view of the genome. Karyotyping gives a broad overview, which FISH refines with its targeted approach. This combination has been instrumental for understanding the genome, aiding accurate diagnosis and treatment, especially in complex cases like hematological malignancies. However, the combined use of FISH and karyotyping has limitations. The primary issue is that many critical aberrations fall below the resolution of karyotyping, even though they are essential for pathogenesis, diagnosis, prognosis, and treatment selection. For example, in AML, there are 16 class-defining aberrations, whereas in B-ALL, this number is 11, according to the 5th edition of the WHO Classification of Haematolymphoid Tumours (WHO-HAEM5) [1,2]. Although FISH panels have been employed to mitigate these limitations, the continually increasing number of crucial aberrations makes their use impractical. Furthermore, emerging biomarkers such as KMT2A-PTD and chromoanagenesis are below the detection resolution of both karyotyping and FISH.

Optical Genome Mapping (OGM) is a cutting-edge technology that is rapidly gaining traction in the field of cytogenetic analysis. It provides a detailed, high-resolution overview of the genome, enabling the identification of a diverse range of structural genomic variations (SVs), including translocations, insertions, inversions, deletions, and duplications. Moreover, OGM is adept at detecting copy number variations (CNVs) and whole-chromosome aneuploidies, consolidating these capabilities into a single comprehensive assay [3,4,5,6,7].

Numerous studies have employed OGM for hematological malignancies, but most of these studies were either in a biomedical research context and/or with a limited sample size of less than 50 cases [8,9,10,11,12,13,14,15,16,17,18,19,20,21,22,23,24,25,26,27,28,29,30], with rare exceptions of studies including approximately 100 cases [31,32,33,34,35,36,37,38,39]. The key difference between research and diagnostic genetics labs is their core objective. Research labs aim to expand our understanding of genetic aberrations in hematological malignancies. In contrast, diagnostic labs prioritize patient benefit, emphasizing the clinical utility of tests. Clinical utility refers to a test’s role in diagnosis, prognosis, and treatment selection [40].

This study aims to bridge a significant gap in our understanding by exploring the application of OGM in a “real-world” clinical laboratory setting, beyond the boundaries of structured research environments. The nature of our study is unique. Unlike exploratory studies that aim to discover novel genetic aberrations in small cohorts or within a specific diagnostic category, our work represents a systematic assessment across all types of hematological malignancies. It focuses on evaluating the added value of OGM compared to SCGW in the context of routine clinical testing. The core question we seek to answer is the added value OGM brings to hematological malignancies beyond SCGW. We are particularly interested in determining whether this value varies among the different types of hematological malignancies. Our investigation centers on two aspects: analytical sensitivity and clinical utility.

Concrete clinical improvements resulting from the use of OGM include the detection of actionable or class-defining aberrations that are missed by a standard cytogenomic workup (SCGW). For instance, OGM uniquely identifies *NUP98* rearrangements in AML, an alteration that may qualify a patient for menin inhibitor therapy. It also detects *MECOM* rearrangements, which are well-known class-defining cytogenetics and adverse prognostic markers in AML, as well as *MEF2D* rearrangements in B-ALL, a class-defining lesion with diagnostic and treatment implications. In T-ALL, OGM uncovers *TLX3* rearrangements that define specific molecular subtypes with prognostic and therapeutic relevance.

Through this exploration, we hope to shed light on the advantages and practicalities of incorporating OGM into standard clinical evaluations of hematological malignancies, broadening the perspective beyond the current research-focused paradigm.

## 2. Materials and Methods

### 2.1. Patients

Our study cohort comprised 519 cases that were tested by the Clinical Cytogenetics Laboratory at MD Anderson Cancer Center, Houston, TXbetween November 2022 and November 2023. There were 207 patients with acute myeloid leukemia (AML), 68 with acute lymphoblastic leukemia (47 B-ALL, 21 T-ALL), 7 with mixed phenotype acute leukemia (MPAL), 87 with myelodysplastic syndrome (MDS), 32 with myeloproliferative neoplasm (MPN), 32 with MDS/MPN, 14 with chronic lymphocytic leukemia (CLL), 24 with mantle cell lymphoma (MCL), 18 with B-Cell Lymphoma other than CLL and MCL cases (“Other BCL”), 15 with multiple myeloma, and 15 with mature T-cell leukemia/lymphoma simultaneously analyzed using OGM and SCGW. Clinicopathologic and laboratory data were gathered through an electronic medical chart review. This study received approval from the Institutional Review Board and adhered to the guidelines of the Declaration of Helsinki.

### 2.2. Chromosomal Analysis

Conventional G-banded chromosomal analysis was routinely performed on bone marrow (BM) aspirate and/or peripheral blood (PB) cultured cells: unstimulated 24-h and 48-h cultures for myeloid neoplasms, or a 72-h culture with mitogen (IL2 and Oligo nucleotides for B-cells and PHA for T-cells) along with a 24-h culture without mitogen for B- or T-cell neoplasms, using standard techniques [41]. A total of 20 metaphases were analyzed for each case, and the findings were reported according to the International System for Human Cytogenetics Nomenclature (ISCN 2020) [42].

### 2.3. Fluorescence In Situ Hybridization

FISH was carried out on BM or PB cultured cells or smears, following the methods previously described [43]. For newly diagnosed acute leukemia patients, a fast FISH screen was routinely conducted, including *CBFB* and *RUNX1T1::RUNX1* for AML, and *BCR::ABL1* for B-ALL and MPAL. In cases of CLL, a FISH panel (*ATM*, *TP53*, *CEP12*, *D13S139*, and *LAMP1)* was performed; for MCL, FISH tests often included *IGH::CCND1*, *MYC* and CLL panel; the myeloma FISH panel included eight FISH tests: *CDK1SB/CKN2C*, *MYC*, *CDKN2A/CEP9*, *IGH::FGFR3*, *IGH::CCND1*, *IGH::MAF*, *RB1/13q34*, and *TP53/CEP17*; FISH for *TCL1A* was specifically performed for patients with a diagnosis of T-PLL.

### 2.4. OGM Analysis

OGM was performed on peripheral blood (PB) or bone marrow (BM) aspirates using the procedures described previously [34,35,37]. All cases in this study underwent triage prior to OGM testing. We used a 20% neoplastic content cut-off to determine whether OGM should be performed in cases of acute leukemia, lymphoma, and multiple myeloma. Briefly, ultra-high molecular weight (UHMW) genomic DNA (gDNA) was extracted from approximately 1.5 million nucleated cells following the manufacturer’s protocols (Bionano Prep SP-G2 DNA Isolation Kit, Catalog# 90151; Bionano Genomics, San Diego, CA, USA); 750 ng UHMW gDNA was applied for a sequence-specific direct label and stain (Bionano Prep DLS-G2 Labeling Kit, Catalog# 80046, San Diego, CA, USA). The purified, fluorescence-labeled gDNA molecules were loaded on a Saphyr G3.3 Chip and then linearized and imaged through massively parallel nanochannel arrays in the Saphyr instrument. The Bionano Access software (version 1.7) was employed for data analysis, utilizing the rare variant analysis pipeline and the Genome Reference Consortium GRCh38/hg38 as the reference genome. The analysis was conducted in two steps, applying two sets of feature files, HemeTargets and hg38-primary_transcripts, along with their corresponding filters. The HemeTargets feature file is custom-designed and encompasses over 500 genes, loci, and fusion genes pertinent to hematologic malignancies. This file was constructed in accordance with guidelines by WHO-HAEM5 [1,2], the International Consensus Classification (ICC) [44,45], the National Comprehensive Cancer Network (NCCN) [46,47,48,49], and the National Health Service (NHS) of the UK. Using this file, we screened for critical SVs or CNVs by using the manufacturer’s recommended confidence level without imposing a minimum size restriction. Concurrently, a 200 Kbp overlap precision was applied to capture gene rearrangements for genes with wide and variable breakpoints. After the initial “hotspot” screening, we shifted to using the hg38-primary_transcripts feature file. For this step, a minimum size of 500 Kbp was set as the filter for both SVs (comprising insertions, deletions, inversions, or duplications) and CNVs.

### 2.5. Variants Interpretation

OGM results were classified according to the 2019 American College of Medical Genetics and Genomics and Clinical Genome Resource (ClinGen) guidelines [50] into three tiers: Tier 1 variants were SVs or CNVs with established diagnostic, prognostic, or therapeutic relevance. These variants are acknowledged in clinical practice guidelines (e.g., National Comprehensive Cancer Network (NCCN), MDS International Prognostic Scoring System, International Myeloma Working Group Criteria), are defined by WHO-HAEM5 or ICC criteria, or are the target of FDA-approved treatments. Tier 2 variants have some clinical relevance but do not satisfy all the criteria for Tier 1. Chromoanagenesis, large CNVs, *KMT2A*-partial tandem duplication (PTD) are examples of Tier 2 variants. Tier 3 variants are acquired aberrations without a known link to neoplastic disorders. Variants that are not eligible for Tier 1 or Tier 2 and cannot be deemed constitutionally benign or likely benign are assigned to Tier 3. These are considered acquired variants of uncertain clinical significance with no established neoplastic association.

### 2.6. Assessment of OGM’s Additional Value

The value of OGM is assessed by comparing OGM findings to those of SCGW in two key aspects: (1) analytical sensitivity—defined as all additional somatic aberrations ((Tier 1, Tier 2, and Tier 3 SVs and CNVs) detected exclusively by OGM; (2) clinical utility—defined as Tier 1 SVs/CNVs detected solely by OGM, which have diagnostic, prognostic, or therapeutic significance.

### 2.7. Statistical Analysis

Minitab software (version 18) was utilized for the statistical analyses. Fisher’s exact test and/or Chi-square test were employed for categorical variables. A *p*-value less than 0.05 was considered statistically significant.

## 3. Results

### 3.1. OGM Enhances Analytical Sensitivity over SCGW

**Figure 1:**
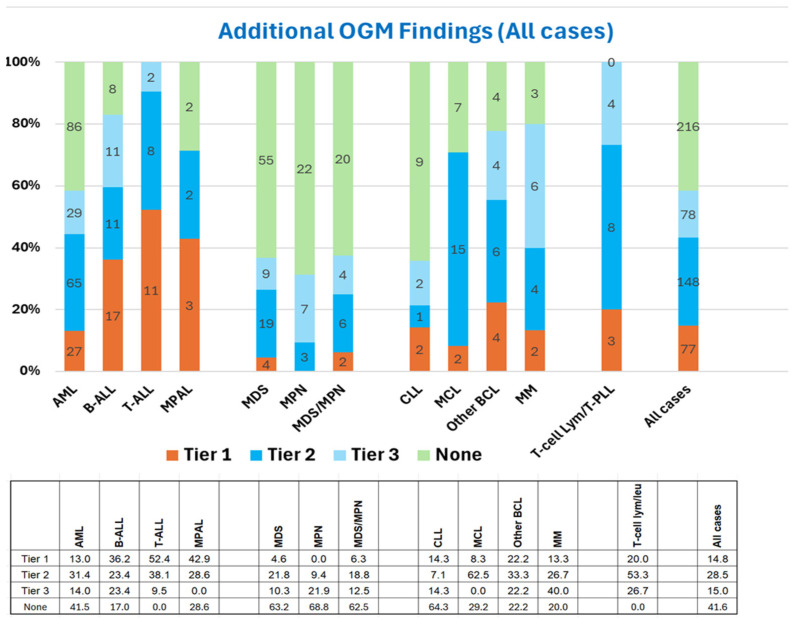
Frequency of additional findings detected using optical genome mapping in different types of hematologic malignancies. The findings are categorized based on clinical relevance, Tier 1 (red), Tier 2 (dark blue), Tier 3 (light blue), and None (green; no additional findings). Numerical values within the bars indicate the number of cases while the table below represent the percentage of cases in each category. AML (acute myeloid leukemia), B-ALL (B-lymphoblastic leukemia), T-ALL (T-lymphoblastic leukemia), MPAL (mixed phenotype acute leukemia), MDS (myelodysplastic syndromes), MPN (myeloproliferative neoplasms), MDS/MPN (myelodysplastic/myeloproliferative neoplasms), CLL (chronic lymphocytic leukemia), MCL (mantle cell lymphoma), BCL (B-cell lymphomas), MM (multiple myeloma), and mature T-cell leukemia/lymphomas.

OGM revealed additional aberrations (including all Tier 1, Tier 2, and Tier 3 SVs and CNVs) in 303 of 519 cases (58%). This increased detection rate varied significantly across hematological malignancies, with additional findings ranging from 31% to 100% of cases (*p* < 0.001). The lowest increase in analytical sensitivity was in MPN cases, while all cases with T-cell malignancies (T-ALL and T-cell lymphoma/leukemia) showed additional findings (Figure 1).

Furthermore, cases with a normal karyotype (41%) showed significantly fewer additional findings than those with an abnormal karyotype (68%) (*p* < 0.001). This is attributable to the differences in myeloid malignancies. Specifically, for AML, the additional findings were seen in 70% in cases with an abnormal karyotype vs. in 30% in cases with a normal karyotype (*p* < 0.001); similar findings were also found in MDS, 44% vs. 17% (*p* < 0.001). Other malignancies did not show statistically significant differences (Supplementary Figure S1A,B).

### 3.2. OGM Enhances the Clinical Utility of SCGW

**Figure 2:**
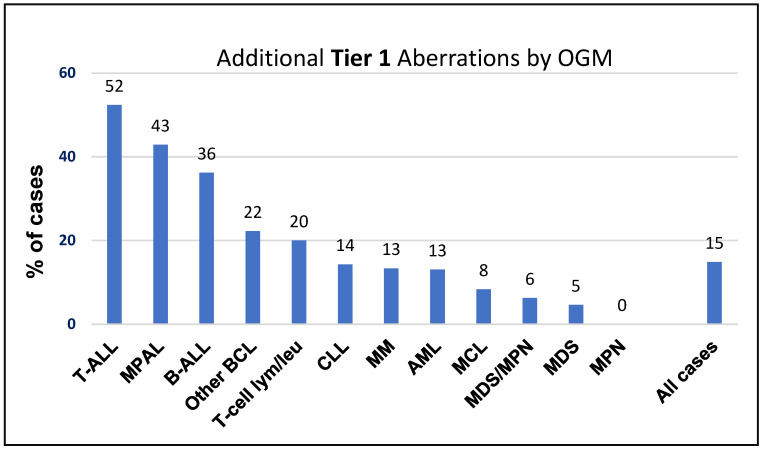
Additional Tier 1 cytogenomic aberrations exclusively detected by optical genome mapping across various hematological malignancies.

OGM identified additional Tier 1 variants in 75 of 519 cases (15%), with detection rates varying by disease type, ranging from 0% in MPN to 52% in T-ALL. Notably, more than 30% of B-ALL, T-ALL, and MPAL cases harbored Tier 1 aberrations that were exclusively detected by OGM (Figure 2).

The Tier 1 aberrations that were not detected by SCGW but identified exclusively by OGM are summarized in Table 1 and are listed in Supplemental Table S1. In AML, rearrangements of *MECOM*, *KMT2A*, and *NUP98* are the most common aberrations missed by SCGW. These three genes had multiple partner genes involved in the rearrangements; some of them were typically cryptic on chromosomal analysis. Classic *MECOM* rearrangement, t(3;3) or inv(3) leading to *GATA2::MECOM*, is often detected by both SCGW and OGM; however, other rearrangement partners, including *MYC*, *CDK6*, *MYB*, *RUNX1*, and *ANGPT1*, may be easily missed by SCGW and are only detected by OGM. The *KMT2A* translocation with partners of *MLLT3*, *AFDN* and *MLLT10* could be missed by SCGW. *NUP98* rearrangements with partner genes such as *HOXA9* and *NSD1* were often cryptic to karyotyping.

For other myeloid neoplasms, *MECOM* rearrangements are the most common additional aberrations detected by OGM. Of note, due to the detection of class-defining gene rearrangements, *MECOM* and *NUP98*, four cases initially diagnosed as MDS were reclassified to AML, based on ICC and WHO-HAEM5 classifications. In addition, critical aberrations, such as rearrangements of *PDGFRA*, *NUP98*, *SYK*, and *NPM1*, were also observed. (Table 1)

Among Ph-negative B-ALL, the clinically impactful aberrations exhibited a wide range of diversity; two or more cases exhibited *ZNF384*, *LYN* and *CRLF2* rearrangements, *PAX5alt*, and *IKZF1* deletions. 7p/*IKZF1* deletion is often small and cryptic on karyotyping.

Rearrangements involving *BCL11B*, including *BCL11B::TLX3*, are the most commonly undetectable Tier 1 aberrations by SCGW in T-ALL (Table 1).

For lymphomas, there is not a predominant aberration. Rare, but highly critical aberrations were detected in individual cases. Examples include *TRA::MTCP1* for T-*PLL*, *TYK2* rearrangement for T-cell lymphomas; *CCND1* and *CCND2* rearrangement with *IGK* and *IGL* in mantle cell lymphoma; *MYC* rearrangements and hyperdiploidy in multiple myeloma (Table 1).

**Figure 3:**
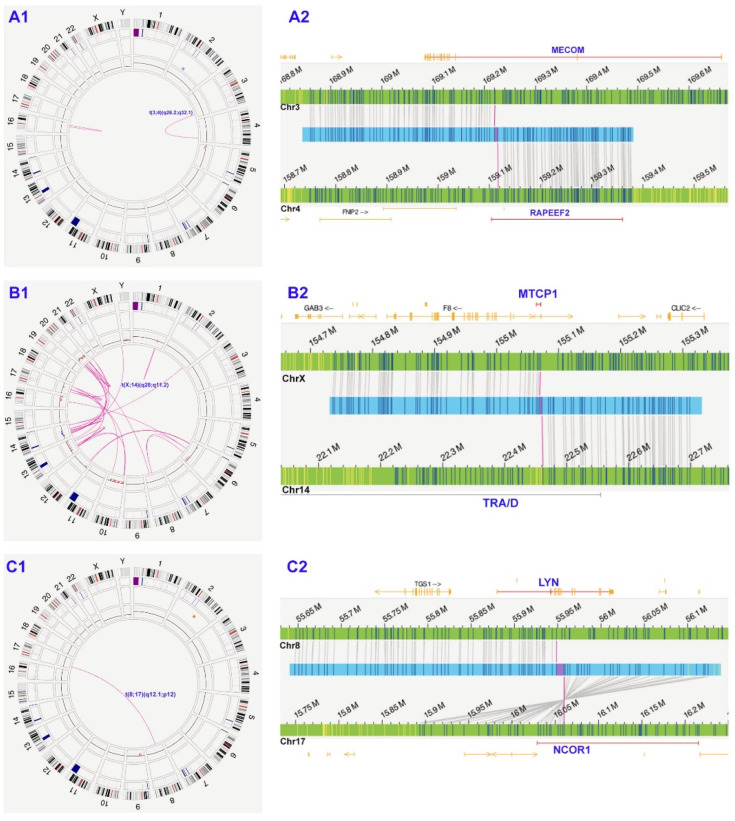
Representative cases exemplifying the clinical utility of the OGM. (**A**) (case #24): initially diagnosed as MDS was reclassified as AML; (**B**) (case #15): T-PLL; (**C**) (case #95): B-ALL. (**A1**,**B1**,**C1**): Circos plots of optical genome mapping outlining the abnormalities; (**A2**,**B2**,**C2**): Genome browser views providing detailed visualization of clinically significant translocations. (**A1**). t(3;4)(q26.2;q31) and intrachromosomal fusion at 16q. (**A2**). t(3;4)(q26.2;q31) leads to *MECOM::RAPEEF2*. (**B1**). Highly complex genome including t(X;14)(q28; q11.2), chromoanagenesis involving chromosomes 14, 14, 17 and 20, copy number losses at 6q, 10p, 10q, 13q, 16q and 20q, and gains at 14q and 20q. (**B2**). t(X;14)(q28; q11.2) resulting in *TRA::MTCP1*. (**C1**). t(8;17)(q12.1;p12) and copy number loss at 9p. (**C2**). t(X;14)(q28; q11.2) resulting in *NCOR1::LYN*.

The aberrations missed by SCGW were often attributed to the following: (1) cryptic rearrangements undetectable by karyotyping (Figure 3A); (2) failure of neoplastic cells to grow, combined with the lack of available FISH probes (Figure 3B); (3) unknown fusion genes (Figure 3C).

**Figure 4:**
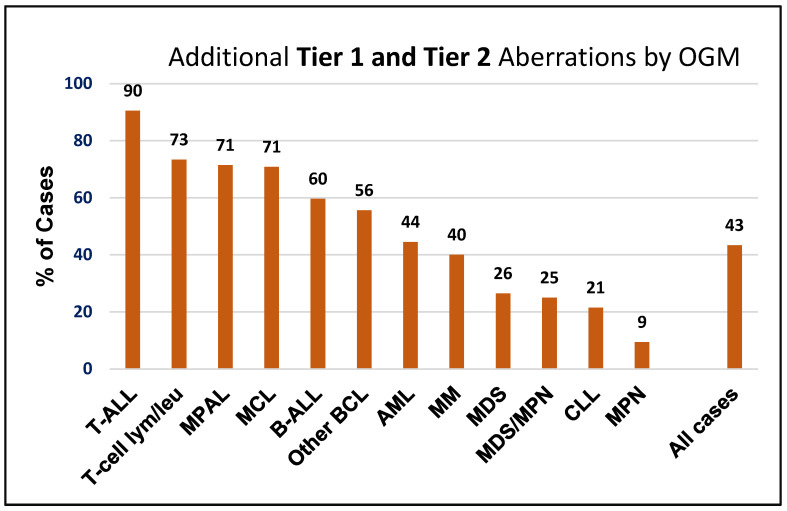
Additional Tier 1 and Tier 2 cytogenomic aberrations detected by optical genome mapping across various hematological malignancies.

When considering both Tier 1 and Tier 2 variants, OGM detected additional variants in 225 of 519 cases (43%), with detection rates ranging from 9% in MPN to 90% in T-ALL. Furthermore, more than 50% of cases with T-ALL, mature T-cell lymphoma/leukemia, MPAL, MCL, B-ALL, and other B-cell lymphomas harbored Tier 1 and Tier 2 aberrations that were exclusively detected by OGM (Figure 4).

Tier 2 variants detected exclusively by OGM are listed in Table 2. Chromoanagenesis, characterized by extensive structural and copy number alterations, was observed across various hematological malignancies, except in MPN and MDS/MPN, and was often associated with highly complex karyotypes. *KMT2A*-PTD, which is cryptic on conventional chromosome and FISH analysis, was identified only in AML, MDS, and MDS/MPN. Additionally, *RUNX1* and *ETV6* rearrangements involving partner genes not classified as class-defining cytogenetic abnormalities were designated as Tier 2 variants in this study. Please see details in Table 2.

## 4. Discussion

The primary objective of this study was to evaluate the extent to which OGM enhances cytogenetic analysis compared to SCGW in hematological malignancies. Additionally, we aimed to determine whether the degree of improvement varied by malignancy type. First, we assessed OGM’s ability to detect additional genetic abnormalities (analytical sensitivity). Second, we examined whether these newly identified abnormalities carried clinical significance for specific hematological malignancies (clinical utility).

Analytical sensitivity refers to OGM’s ability to detect additional SVs or CNVs beyond those identified by SCGW. As expected, OGM identified more aberrations in approximately two-thirds of cases. However, the frequency of additional findings varied by disease type, with the lowest detection rates in MPN and MDS/MPN and the highest in T-cell malignancies. An interesting observation is the difference in the added value of OGM between myeloid malignancies with normal versus abnormal karyotypes. In most cases, samples with a normal karyotype remain normal even after OGM analysis. This suggests that the primary benefit of OGM lies in its ability to detect critical aberrations within complex karyotypes, rather than uncovering cryptic cytogenetic abnormalities in cases with an apparently normal karyotype. However, a notable exception is *KMT2A*-PTD, which is often presented in cases with a normal karyotype.

OGM offers several key analytical advantages. First, it reduces false negatives that may arise due to the predominance of normal cells in tissue cultures, which is particularly beneficial for analyzing mature B- and T-cell malignancies with low mitotic activity. Second, as a genome-wide assay, OGM provides broader coverage compared to targeted FISH panels. Third, its higher resolution allows for the detection of cryptic aberrations and small duplications that are often missed by SCGW, such as *KMT2A* partial tandem duplications. Finally, its enhanced resolution aids in identifying novel gene fusions, contributing to more precise disease classification and improved diagnostic accuracy.

OGM has its own analytical limitations. Unlike single-cell assays such as karyotyping and FISH, OGM is a bulk DNA assay, which means it produces results comparable to composite karyotypes, limiting direct observation of clonal architecture. Another limitation is its limit of detection (LOD). With an LOD of 20%, OGM is significantly less sensitive than karyotyping and FISH, making it challenging to detect aberrations in specimens with low-level neoplastic cell involvement. Additionally, OGM requires ultra-high molecular weight DNA and a minimum of 15 micrograms of genomic DNA, restricting its clinical applicability in formalin-fixed paraffin-embedded (FFPE) tissues and hypocellular specimens.

While enhanced analytical sensitivity is essential, it is not sufficient for implementing a new genetic assay in clinical practice. The primary rationale for integrating OGM into clinical laboratory workflows is its ability to detect Tier 1 abnormalities with direct clinical relevance.

An example of a Tier 1 abnormality is *NUP98* rearrangement in AML—a class-defining cytogenetics alteration—where detection of *NUP98*-R qualifies patients for treatment with menin inhibitors. In contrast, recurrent chromosome 3q gain in MCL is an example of a Tier 2 variant. Although frequently observed, it lacks established clinical utility for diagnosis or management of MCL. For some variants, however, the distinction between Tier 1 and Tier 2 is less clear-cut, and classification may be somewhat subjective. In this study, we classified chromoanagenesis and *KMT2A*-PTD as Tier 2 aberrations. While chromoanagenesis has been suggested as an adverse prognostic factor [34,51,52], it is not yet recognized as a prognosis-defining biomarker in clinical guidelines. Although *KMT2A*-PTD has been recognized as a high-risk molecular feature in the International Prognostic Scoring System-Molecular (IPSS-M) in MDS [53], its prognostic significance in AML remains controversial [35,54].

In AML and MDS, *KMT2A* and *MECOM* rearrangements are the most common aberrations missed by SCGW but detected by OGM. Both genes have multiple translocation partners, some of which are cryptic or subtle, making them difficult to detect by karyotyping. OGM also identified critical rearrangements involving *NUP98*, *JAK2*, *FGFR1*, and *RUNX1* (excluding *RUNX1::RUNXT1*). Interestingly, most *MECOM* rearrangements missed by karyotyping were non-classical variants rather than the typical inv(3)(q21;q26.2), suggesting that these aberrations may be underrecognized by karyotyping. Overall, the frequency of additional clinically significant aberrations detected by OGM in this study aligns with previously reported findings in myeloid malignancies [3,9,22,31,55]

In Ph-negative B-ALL, the clinically impactful aberrations exhibit a wide range of diversity. The presence of low-frequency but highly impactful aberrations, such as *LYN* or *ZNF384* rearrangements, underscores the significance of using OGM for Ph-negative B-ALL. In the context of B-ALL, it is crucial not to miss the long tail of clinically relevant aberrations. Our findings align with earlier OGM studies conducted in B-ALL [11,12,14,19,25].

In T-ALL and MPAL, approximately 60% of cases exhibit Tier 1 abnormalities. Although T-ALL and MPAL are relatively rare, they are characterized by their high aggressiveness. In T-ALL, *BCL11B* rearrangements serve as the primary driver for classification, yet nearly half of T-ALL cases also display other class-defining critical aberrations. The detection of *RUNX1* and *BCL11B* rearrangements in different cases of MPAL, the M/T subtype, highlights the ongoing genomic heterogeneity within MPAL.

In B-cell lymphomas, the big advantage of OGM is its ability to detect disease-related cytogenomic changes that may be difficult to detect by karyotyping due to the poor or non-growth of neoplastic cells. For example, up to 50% of MCL cases lack informative karyotypic data, whereas OGM successfully detected cytogenomic abnormalities in all MCL cases. Another advantage of OGM in B-cell lymphomas is its ability to identify uncommon translocations, particularly those involving *IGK* or *IGL* loci. For example, in MCL, where conventional testing has primarily focused on *IGH::CCND1*, OGM enables the detection of other clinically relevant translocations, such as *IGL::CCND2* or *IGK::CCND1*

This study has several limitations. First, as a single-institution study at a major academic center focused on adult malignancies, its findings may not be generalizable to community oncology centers or pediatric hospitals with a different patient mix. Second, differences in SCGW workup, particularly FISH studies, may affect the applicability of our results to other laboratories. Third, the classification of clinically relevant aberrations (Tier 1 and Tier 2) remains somewhat subjective, with potential variability among cytogeneticists. Fourth, our assessment of the clinical impact of certain cytogenetic aberrations reflects current understanding but may evolve over time. Lastly, this study evaluates OGM’s value relative to karyotyping and FISH without considering other assays such as NGS-based fusion panels, whole genome sequencing, or array-based copy number analysis.

## 5. Conclusions

The integration of OGM with SCGW provides significant benefits for patient management by enhancing the detection of clinically relevant cytogenomic aberrations. However, its impact varies across different types of hematological malignancies. Given these differences, laboratories should carefully consider the analytical advantages, clinical relevance, and cost-effectiveness of OGM within their specific testing workflows. Strategically incorporating OGM alongside conventional cytogenetic methods can optimize diagnostic accuracy, prognostic assessment, and personalized treatment strategies, ultimately improving patient outcomes.

## Figures and Tables

**Table 1:** Tier 1 aberrations exclusively detected by OGM, not by SCGW.

	Disease	AML	B-ALL	T-ALL	MPAL	MDS	MPN	MDS/MPN	CLL	MCL	Other BCL	MM	T-Cell lym	Total
Aberrations														
*MECOM-R*		11				3								14
*KMT2A-R*		5		1										6
*NUP98-R*		5						1						6
*DEK::NUP214*		1												1
*NPM1::MLF1*						1								1
*CBFA2T3::GLIS2*		1												1
*JAK2-R*		2	1											3
*FGFR1-R*		1												1
del(5q)		1												1
*LYN-R*			2											2
*SYK*								1						1
*IGH::IL3*			1											1
*ETV6::RUNX1*			1											1
*PAX5alt*			2											2
*MEF2D-R*			1											1
*ZNF384-R*			2											2
*IKZF1* ^loss^			6											6
*MLLT10::PICALM*			1	1	1									3
*BCL11B-R*				3	1									4
*TLX3-R*				3										3
*HOXA3::TCRB*				1										1
*NUP214::ABL1*				1	1									2
Complex karyotype									2					2
*CCND1-R*										1				1
*CCND2-R*										1	1			2
*BCL2-R*											1			1
*del(7q)*											1			1
*MYC*-R											1	2		3
*MTCP1-R*													2	2
**Total**		27	17	10	3	4	0	2	2	2	4	2	2	75

**Table 2:** Tier 2 aberrations exclusively detected by OGM, not by SCGW.

	Disease	AML	B-ALL	T-ALL	MPAL	MDS	MPN	MDS/MPN	CLL	MCL	Other BCL	MM	T-Cell lym	Total
Aberrations														
*Chromoanagenesis*		32	5	5		12				5	3	2	3	67
*KMT2A PTD*		14			1	2		2						19
*CNVs >=5Mb*		7	2	2		1	2	2	1	10	3		5	35
*RUNX1-R*		4			1	2								7
*ETV6-R*		2						1						3
*KMT2A amp*		2				1								3
*TET2 loss*		1				1	1	1						4
*VDR::CBF2AT3*		1												1
*MECOM amp*		1												1
*CDKN2A/B del*		1	4											5
*MYB-R*				1										1
*TRA-R*				1										1
*Hyperdiploidy*												1		1
*MYC amp*												1		1
*TYK2-R*													1	1
Total		65	11	9	2	19	3	6	1	15	6	4	9	150

## Data Availability

The original contributions presented in this study are included in the article/Supplementary Material. Further inquiries can be directed to the corresponding author.

## Supplementary Materials

The following supporting information can be downloaded at: https://www.mdpi.com/article/10.3390/cancers17091436/s1, Figure S1: (A). Additional OGM Findings in Cases with Abnormal Karyotype; (B). Additional OGM Findings in Cases with Normal Karyotype Table S1: Case Level Data.

## References

[1] Alaggio R., Amador C., Anagnostopoulos I., Attygalle A.D., Araujo I.B.O., Berti E., Bhagat G., Borges A.M., Boyer D., Calaminici M. (2022). The 5th edition of the World Health Organization Classification of Haematolymphoid Tumours: Lymphoid Neoplasms. Leukemia.

[2] Khoury J.D., Solary E., Abla O., Akkari Y., Alaggio R., Apperley J.F., Bejar R., Berti E., Busque L., Chan J.K.C. (2022). The 5th edition of the World Health Organization Classification of Haematolymphoid Tumours: Myeloid and Histiocytic/Dendritic Neoplasms. Leukemia.

[3] Coccaro N., Anelli L., Zagaria A., Tarantini F., Cumbo C., Tota G., Minervini C.F., Minervini A., Conserva M.R., Redavid I. (2023). Feasibility of Optical Genome Mapping in Cytogenetic Diagnostics of Hematological Neoplasms: A New Way to Look at DNA. Diagnostics.

[4] Garcia-Heras J. (2021). Optical Genome Mapping: A Revolutionary Tool for “Next Generation Cytogenomics Analysis” with a Broad Range of Diagnostic Applications in Human Diseases. J. Assoc. Genet. Technol..

[5] Sahajpal N.S., Mondal A.K., Hastie A., Chaubey A., Kolhe R. (2023). Optical Genome Mapping for Oncology Applications. Curr. Protoc..

[6] Smith A.C., Neveling K., Kanagal-Shamanna R. (2022). Optical genome mapping for structural variation analysis in hematologic malignancies. Am. J. Hematol..

[7] Balciuniene J., Ning Y., Lazarus H.M., Aikawa V., Sherpa S., Zhang Y., Morrissette J.J. (2024). Cancer cytogenetics in a genomics world: Wedding the old with the new. Blood Rev..

[8] Balducci E., Kaltenbach S., Villarese P., Duroyon E., Zalmai L., Friedrich C., Suarez F., Marcais A., Bouscary D., Decroocq J. (2022). Optical genome mapping refines cytogenetic diagnostics, prognostic stratification and provides new molecular insights in adult MDS/AML patients. Blood Cancer J..

[9] Gerding W.M., Tembrink M., Nilius-Eliliwi V., Mika T., Dimopoulos F., Ladigan-Badura S., Eckhardt M., Pohl M., Wünnenberg M., Farshi P. (2022). Optical genome mapping reveals additional prognostic information compared to conventional cytogenetics in AML/MDS patients. Int. J. Cancer.

[10] Jean J., Kovach A.E., Doan A., Oberley M.J., Ji J., Schmidt R.J., Biegel J.A., Bhojwani D., Raca G. (2022). Characterization of *PAX5* intragenic tandem multiplication in pediatric B-lymphoblastic leukemia by optical genome mapping. Blood Adv..

[11] Lestringant V., Duployez N., Penther D., Luquet I., Derrieux C., Lutun A., Preudhomme C., West M., Ouled-Haddou H., Devoldere C. (2021). Optical genome mapping, a promising alternative to gold standard cytogenetic approaches in a series of acute lymphoblastic leukemias. Genes Chromosom. Cancer.

[12] Lühmann J.L., Stelter M., Wolter M., Kater J., Lentes J., Bergmann A.K., Schieck M., Göhring G., Möricke A., Cario G. (2021). The Clinical Utility of Optical Genome Mapping for the Assessment of Genomic Aberrations in Acute Lymphoblastic Leukemia. Cancers.

[13] Puiggros A., Ramos-Campoy S., Kamaso J., de la Rosa M., Salido M., Melero C., Rodríguez-Rivera M., Bougeon S., Collado R., Gimeno E. (2022). Optical Genome Mapping: A Promising New Tool to Assess Genomic Complexity in Chronic Lymphocytic Leukemia (CLL). Cancers.

[14] Rack K., De Bie J., Ameye G., Gielen O., Demeyer S., Cools J., De Keersmaecker K., Vermeesch J.R., Maertens J., Segers H. (2022). Optimizing the diagnostic workflow for acute lymphoblastic leukemia by optical genome mapping. Am. J. Hematol..

[15] Ramos-Campoy S., Puiggros A., Kamaso J., Beà S., Bougeon S., Larráyoz M.J., Costa D., Parker H., Rigolin G.M., Blanco M.L. (2022). *TP53* Abnormalities Are Underlying the Poor Outcome Associated with Chromothripsis in Chronic Lymphocytic Leukemia Patients with Complex Karyotype. Cancers.

[16] Suttorp J., Lühmann J.L., Behrens Y.L., Göhring G., Steinemann D., Reinhardt D., von Neuhoff N., Schneider M. (2022). Optical Genome Mapping as a Diagnostic Tool in Pediatric Acute Myeloid Leukemia. Cancers.

[17] Giguère A., Raymond-Bouchard I., Collin V., Claveau J.-S., Hébert J., LeBlanc R. (2023). Optical Genome Mapping Reveals the Complex Genetic Landscape of Myeloma. Cancers.

[18] Díaz-González Á., Mora E., Avetisyan G., Furió S., De la Puerta R., Gil J.V., Liquori A., Villamón E., García-Hernández C., Santiago M. (2023). Cytogenetic Assessment and Risk Stratification in Myelofibrosis with Optical Genome Mapping. Cancers.

[19] Gao H., Xu H., Wang C., Cui L., Huang X., Li W., Yue Z., Tian S., Zhao X., Xue T. (2022). Optical Genome Mapping for Comprehensive Assessment of Chromosomal Aberrations and Discovery of New Fusion Genes in Pediatric B-Acute Lymphoblastic Leukemia. Cancers.

[20] Kriegova E., Fillerova R., Minarik J., Savara J., Manakova J., Petrackova A., Dihel M., Balcarkova J., Krhovska P., Pika T. (2021). Whole-genome optical mapping of bone-marrow myeloma cells reveals association of extramedullary multiple myeloma with chromosome 1 abnormalities. Sci. Rep..

[21] Neveling K., Mantere T., Vermeulen S., Oorsprong M., van Beek R., Kater-Baats E., Pauper M., van der Zande G., Smeets D., Weghuis D.O. (2021). Next-generation cytogenetics: Comprehensive assessment of 52 hematological malignancy genomes by optical genome mapping. Am. J. Hum. Genet..

[22] Soler G., Ouedraogo Z.G., Goumy C., Lebecque B., Requena G.A., Ravinet A., Kanold J., Véronèse L., Tchirkov A. (2023). Optical Genome Mapping in Routine Cytogenetic Diagnosis of Acute Leukemia. Cancers.

[23] Valkama A., Vorimo S., Kumpula T.A., Räsänen H., Savolainen E.-R., Pylkäs K., Mantere T. (2023). Optical Genome Mapping as an Alternative to FISH-Based Cytogenetic Assessment in Chronic Lymphocytic Leukemia. Cancers.

[24] Vangala D.B., Nilius-Eliliwi V., Gerding W.M., Schroers R., Nguyen H.P. (2022). Optical Genome Mapping in MDS and AML as tool for structural variant profiling—Comment and data update on Yang et al.: “High-resolution structural variant profiling of myelodysplastic syndromes by optical genome mapping uncovers cryptic aberrations of prognostic and therapeutic significance”. Leukemia.

[25] Vieler L.-M., Nilius-Eliliwi V., Schroers R., Ben Vangala D., Nguyen H.P., Gerding W.M. (2023). Optical Genome Mapping Reveals and Characterizes Recurrent Aberrations and New Fusion Genes in Adult ALL. Genes.

[26] Valkama A., Vorimo S., Tervasmäki A., Räsänen H., Savolainen E., Pylkäs K., Mantere T. (2025). Structural Variant Analysis of Complex Karyotype Myelodysplastic Neoplasia Through Optical Genome Mapping. Genes Chromosom. Cancer.

[27] Xu H., Gao H., Wang C., Cheng X., Li Z., Lei C., Huang X., Li W., Yue Z., Tian S. (2024). Optical Genome Mapping Reveals Novel Structural Variants in Lymphoblastic Lymphoma. J. Pediatr. Hematol..

[28] Seto A., Downs G., King O., Salehi-Rad S., Baptista A., Chin K., Grenier S., Nwachukwu B., Tierens A., Minden M.D. (2024). Genomic Characterization of Partial Tandem Duplication Involving the *KMT2A* Gene in Adult Acute Myeloid Leukemia. Cancers.

[29] Budurlean L., Tukaramrao D.B., Zhang L., Dovat S., Broach J. (2024). Integrating Optical Genome Mapping and Whole Genome Sequencing in Somatic Structural Variant Detection. J. Pers. Med..

[30] Zou Y.S., Klausner M., Ghabrial J., Stinnett V., Long P., Morsberger L., Murry J.B., Beierl K., Gocke C.D., Xian R.R. (2024). A comprehensive approach to evaluate genetic abnormalities in multiple myeloma using optical genome mapping. Blood Cancer J..

[31] Levy B., Baughn L.B., Akkari Y., Chartrand S., LaBarge B., Claxton D., Lennon P.A., Cujar C., Kolhe R., Kroeger K. (2023). Optical genome mapping in acute myeloid leukemia: A multicenter evaluation. Blood Adv..

[32] Yang H., Garcia-Manero G., Sasaki K., Montalban-Bravo G., Tang Z., Wei Y., Kadia T., Chien K., Rush D., Nguyen H. (2022). High-resolution structural variant profiling of myelodysplastic syndromes by optical genome mapping uncovers cryptic aberrations of prognostic and therapeutic significance. Leukemia.

[33] Sahajpal N.S., Mondal A.K., Tvrdik T., Hauenstein J., Shi H., Deeb K.K., Saxe D., Hastie A.R., Chaubey A., Savage N.M. (2022). Clinical Validation and Diagnostic Utility of Optical Genome Mapping for Enhanced Cytogenomic Analysis of Hematological Neoplasms. J. Mol. Diagn..

[34] Wei Q., Hu S., Loghavi S., Toruner G.A., Ravandi-Kashani F., Tang Z., Li S., Xu J., Daver N., Medeiros L.J. (2025). Chromoanagenesis Is Frequently Associated with Highly Complex Karyotypes, Extensive Clonal Heterogeneity, and Treatment Refractoriness in Acute Myeloid Leukemia. Am. J. Hematol..

[35] Wei Q., Hu S., Xu J., Loghavi S., Daver N., Toruner G.A., Wang W., Medeiros L.J., Tang G. (2024). Detection of *KMT2A* Partial Tandem Duplication by Optical Genome Mapping in Myeloid Neoplasms: Associated Cytogenetics, Gene Mutations, Treatment Responses, and Patient Outcomes. Cancers.

[36] Hidalgo-Gómez G., Tazón-Vega B., Palacio C., Saumell S., Martínez-Morgado N., Navarro V., Murillo L., Velasco P., Murciano T., de Heredia C.D. (2025). How to combine multiple tools for the genetic diagnosis work-up of pediatric B-cell acute lymphoblastic leukemia. Ann. Hematol..

[37] Loghavi S., Wei Q., Ravandi F., Quesada A.E., Routbort M.J., Hu S., Toruner G.A., Wang S.A., Wang W., Miranda R.N. (2024). Optical genome mapping improves the accuracy of classification, risk stratification, and personalized treatment strategies for patients with acute myeloid leukemia. Am. J. Hematol..

[38] Levy B., Kanagal-Shamanna R., Sahajpal N.S., Neveling K., Rack K., Dewaele B., Weghuis D.O., Stevens-Kroef M., Puiggros A., Mallo M. (2024). A framework for the clinical implementation of optical genome mapping in hematologic malignancies. Am. J. Hematol..

[39] Lacoste S.A., Gagnon V., Béliveau F., Lavallée S., Collin V., Hébert J. (2024). Unveiling the Complexity of *KMT2A* Rearrangements in Acute Myeloid Leukemias with Optical Genome Mapping. Cancers.

[40] Joseph L., Cankovic M., Caughron S., Chandra P., Emmadi R., Hagenkord J., Hallam S., Jewell K.E., Klein R.D., Pratt V.M. (2016). The Spectrum of Clinical Utilities in Molecular Pathology Testing Procedures for Inherited Conditions and Cancer. J. Mol. Diagn..

[41] Liu W., Burger J.A., Xu J., Tang Z., Toruner G., Khanlari M., Medeiros L.J., Tang G. (2020). LPL deletion is associated with poorer response to ibrutinib-based treatments and overall survival in TP53-deleted chronic lymphocytic leukemia. Ann. Hematol..

[42] McGowan-Jordan J., Hastings R.J., Moore S. (2020). ISCN (2020): An International System for Human Cytogenetic Nomenclature (2020).

[43] Mascarello J.T., Hirsch B., Kearney H.M., Ketterling R.P., Olson S.B., Quigley D.I., Rao K.W., Tepperberg J.H., Tsuchiya K.D., Wiktor A.E. (2011). Section E9 of the American College of Medical Genetics technical standards and guidelines: Fluorescence in situ hybridization. Anesth. Analg..

[44] Arber D.A., Orazi A., Hasserjian R.P., Borowitz M.J., Calvo K.R., Kvasnicka H.-M., Wang S.A., Bagg A., Barbui T., Branford S. (2022). International Consensus Classification of Myeloid Neoplasms and Acute Leukemias: Integrating morphologic, clinical, and genomic data. Blood.

[45] Campo E., Jaffe E.S., Cook J.R., Quintanilla-Martinez L., Swerdlow S.H., Anderson K.C., Brousset P., Cerroni L., de Leval L., Dirnhofer S. (2022). The International Consensus Classification of Mature Lymphoid Neoplasms: A report from the Clinical Advisory Committee. Blood.

[46] Brown P.A., Shah B., Advani A., Aoun P., Boyer M.W., Burke P.W., DeAngelo D.J., Dinner S., Fathi A.T., Gauthier J. (2021). Acute Lymphoblastic Leukemia, Version 2.2021, NCCN Clinical Practice Guidelines in Oncology. J. Natl. Compr. Cancer Netw..

[47] Pollyea D.A., Altman J.K., Assi R., Bixby D., Fathi A.T., Foran J.M., Gojo I., Hall A.C., Jonas B.A., Kishtagari A. (2023). Acute Myeloid Leukemia, Version 3.2023, NCCN Clinical Practice Guidelines in Oncology. J. Natl. Compr. Cancer Netw..

[48] Wierda W.G., Brown J., Abramson J.S., Awan F., Bilgrami S.F., Bociek G., Brander D., Chanan-Khan A.A., Coutre S.E., Davis R.S. (2022). NCCN Guidelines^®^ Insights: Chronic Lymphocytic Leukemia/Small Lymphocytic Lymphoma, Version 3. J. Natl. Compr. Cancer Netw..

[49] Zelenetz A.D., Gordon L.I., Abramson J.S., Advani R.H., Andreadis B., Bartlett N.L., Budde L.E., Caimi P.F., Chang J.E., Christian B. (2023). NCCN Guidelines^®^ Insights: B-Cell Lymphomas, Version 6. J. Natl. Compr. Cancer Netw..

[50] Mikhail F.M., Biegel J.A., Cooley L.D., Dubuc A.M., Hirsch B., Horner V.L., Newman S., Shao L., Wolff D.J., Raca G. (2019). Technical laboratory standards for interpretation and reporting of acquired copy-number abnormalities and copy-neutral loss of heterozygosity in neoplastic disorders: A joint consensus recommendation from the American College of Medical Genetics and Genomics (ACMG) and the Cancer Genomics Consortium (CGC). Anesth. Analg..

[51] Bochtler T., Granzow M., Stölzel F., Kunz C., Mohr B., Kartal-Kaess M., Hinderhofer K., Heilig C.E., Kramer M., Thiede C. (2017). Marker chromosomes can arise from chromothripsis and predict adverse prognosis in acute myeloid leukemia. Blood.

[52] Fontana M.C., Marconi G., Feenstra J.D.M., Fonzi E., Papayannidis C., Di Rorá A.G.L., Padella A., Solli V., Franchini E., Ottaviani E. (2018). Chromothripsis in acute myeloid leukemia: Biological features and impact on survival. Leukemia.

[53] Bernard E., Tuechler H., Greenberg P.L., Hasserjian R.P., Ossa J.E.A., Nannya Y., Devlin S.M., Creignou M., Pinel P., Monnier L. (2022). Molecular International Prognostic Scoring System for Myelodysplastic Syndromes. NEJM Evid..

[54] Ye W., Ma M., Wu X., Deng J., Liu X., Zheng X., Gong Y. (2023). Prognostic significance of *KMT2A*-PTD in patients with acute myeloid leukaemia: A systematic review and meta-analysis. BMJ Open.

[55] Nilius-Eliliwi V., Gerding W.M., Schroers R., Nguyen H.P., Vangala D.B. (2023). Optical Genome Mapping for Cytogenetic Diagnostics in AML. Cancers.

