# Supplementary material for: Clinical Utility of Optical Genome Mapping as an Additional Tool in a Standard Cytogenetic Workup in Hematological Malignancies
Source: Cancers (Basel). 2025 Apr 25;17(9):1436. doi: 10.3390/cancers17091436 (PMC12070923; doi:10.3390/cancers17091436)
Supplement: Supplementary file 1 [file cancers-17-01436-s001.zip › SupplementaryFigureTorunerv8.pptx]

## Slide 1
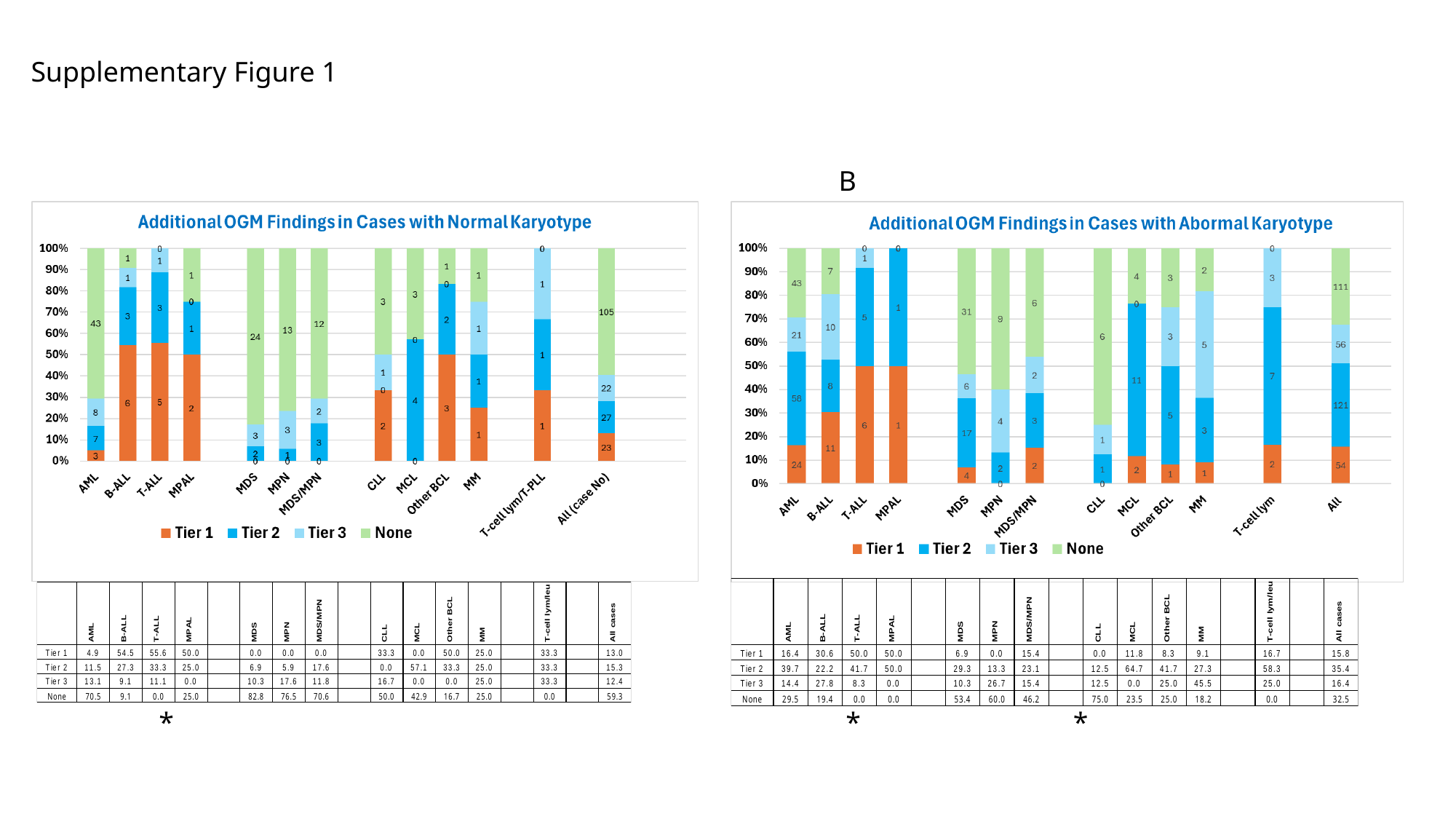

Supplementary Figure 1
A B
* * * *

## Slide 2
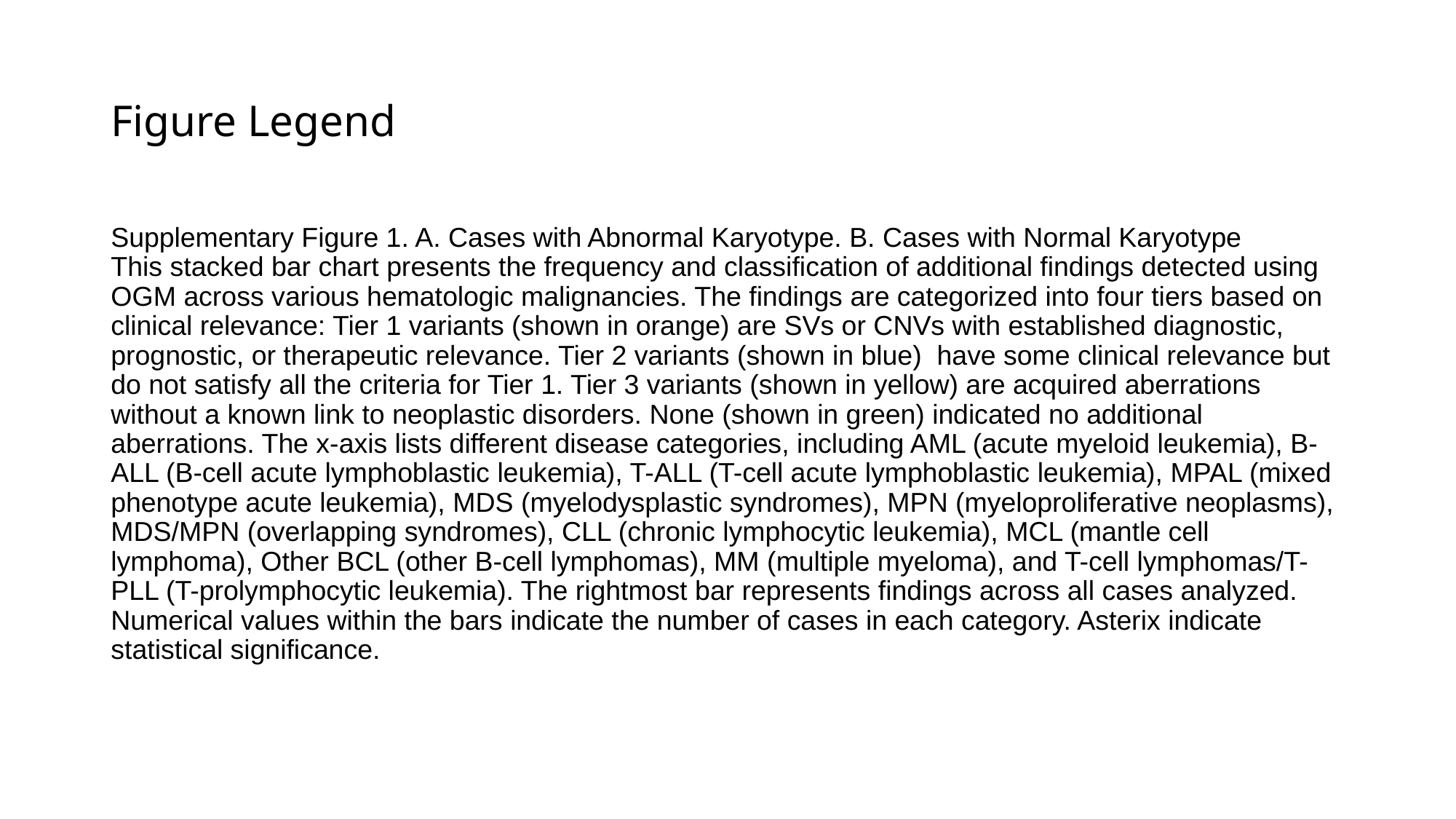

# Figure Legend
Supplementary Figure 1. A. Cases with Abnormal Karyotype. B. Cases with Normal Karyotype
This stacked bar chart presents the frequency and classification of additional findings detected using OGM across various hematologic malignancies. The findings are categorized into four tiers based on clinical relevance: Tier 1 variants (shown in orange) are SVs or CNVs with established diagnostic, prognostic, or therapeutic relevance. Tier 2 variants (shown in blue) have some clinical relevance but do not satisfy all the criteria for Tier 1. Tier 3 variants (shown in yellow) are acquired aberrations without a known link to neoplastic disorders. None (shown in green) indicated no additional aberrations. The x-axis lists different disease categories, including AML (acute myeloid leukemia), B-ALL (B-cell acute lymphoblastic leukemia), T-ALL (T-cell acute lymphoblastic leukemia), MPAL (mixed phenotype acute leukemia), MDS (myelodysplastic syndromes), MPN (myeloproliferative neoplasms), MDS/MPN (overlapping syndromes), CLL (chronic lymphocytic leukemia), MCL (mantle cell lymphoma), Other BCL (other B-cell lymphomas), MM (multiple myeloma), and T-cell lymphomas/T-PLL (T-prolymphocytic leukemia). The rightmost bar represents findings across all cases analyzed. Numerical values within the bars indicate the number of cases in each category. Asterix indicate statistical significance.
